# Diffuse alveolar hemorrhage in children with trisomy 21

**DOI:** 10.1186/s12969-021-00592-4

**Published:** 2021-07-17

**Authors:** Jessica L. Bloom, Benjamin Frank, Jason P. Weinman, Csaba Galambos, Sean T. O’Leary, Deborah R. Liptzin, Robert C. Fuhlbrigge

**Affiliations:** 1 Department of Pediatrics, Section of Pediatric Rheumatology, University of Colorado Anschutz Medical Campus, Aurora, CO USA; 2 Department of Pediatrics, Section of Pediatric Cardiology, University of Colorado, Anschutz Medical Campus, Aurora, CO USA; 3 Department of Radiology, |University of Colorado Anschutz Medical Campus, Aurora, CO USA; 4 Department of Pathology and Laboratory Medicine, University of Colorado Anschutz Medical Campus, Aurora, CO USA; 5 Department of Pediatrics, Section of Infectious Disease, Adult and Child Consortium for Health Outcomes Research and Delivery Science, University of Colorado Anschutz Medical Campus, Aurora, CO USA; 6 Department of Pediatrics, Section of Pediatric Pulmonology and Sleep Medicine, University of Colorado, Anschutz Medical Campus, Aurora, CO USA

**Keywords:** Pulmonary hemorrhage, Diffuse alveolar hemorrhage, Down syndrome, Trisomy 21, Vasculitis, Capillaritis, Autoimmune disease, Hemoptysis, Autoantibodies

## Abstract

**Background:**

Respiratory conditions are the leading cause of hospitalization and death in children with Trisomy 21 (T21). Diffuse alveolar hemorrhage (DAH) occurs at higher frequency in children with T21; yet, it is not widely studied nor is there a standardized approach to diagnosis or management. The objective of this study was to identify children with T21 and DAH in order to understand contributing factors and identify opportunities to improve outcomes. We identified 5 children with T21 at a single institution with histology-proven DAH over 10 years and discuss their presentation, evaluation, management, and outcomes. We also reviewed the cases in the literature.

**Case presentation:**

Patient 1 died at age seven due to secondary hemophagocytic lymphohistiocytosis. DAH was seen on autopsy. Patient 2 was a three-year-old with systemic-onset juvenile idiopathic arthritis diagnosed with DAH after presenting for hypoxia. Patient 3 was diagnosed with DAH at age nine after presenting with recurrent suspected pneumonia and aspiration. Patient 4 was diagnosed with DAH at age eight after presenting with pallor and fatigue. She had additional ICU admissions for DAH with infections. Patient 5 developed hemoptysis at age three and had recurrent DAH for 10 years. Four patients responded positively to immune-modulation such as intravenous immunoglobulin, glucocorticoids, and rituximab. Of the 19 patients identified in the literature, only one was from the United States. The majority had anemia, respiratory distress, autoantibodies, and recurrences. Very few patients had hemoptysis. Idiopathic pulmonary hemosiderosis was the most common diagnosis. Almost all received glucocorticoids with or without additional immunosuppression. The majority of our patients and those in the literature had positive auto-antibodies such as anti-neutrophil cytoplasmic antibodies and anti-nuclear antigen antibodies. Diagnostic clues included respiratory distress, hypoxia, anemia, recurrent pneumonia, and/or ground glass opacities on imaging. We identified four contributors to DAH: structural lung abnormalities, pulmonary arterial hypertension, infection/aspiration, and autoimmune disease/immune dysregulation.

**Conclusion:**

These cases demonstrate the need for an increased index of suspicion for DAH in children with T21, particularly given the low frequency of hemoptysis at presentation, enrich the understanding of risk factors, and highlight the favorable response to immunosuppressive therapies in this vulnerable population.

## Background

Trisomy 21 (T21; or Down Syndrome) results from complete or partial duplication of chromosome 21. T21 affects 1 in 700 births and is characterized by developmental disability and dysmorphic features [1]. Children with T21 are hospitalized five times more often, with respiratory conditions the leading cause of hospitalization and mortality [1–5]. Diffuse alveolar hemorrhage (DAH) is a life-threatening cause of respiratory compromise that occurs at increased frequency in children with T21.

DAH occurs when blood enters the alveolar spaces due to a disturbance of the alveolar-capillary basement membrane and can result in respiratory failure and death [6]. The diagnosis may be delayed or missed in children, as many do not expectorate blood and have non-specific findings on imaging [7]. In one study, two-thirds of children with DAH were initially misdiagnosed, most often with pneumonia [8]. Definitive diagnosis is made via bloody fluid return, microscopic red blood cells, or hemosiderin-laden macrophages on bronchoalveolar lavage (BAL) or alveolar hemorrhage or hemosiderin on lung histology [9]. Treatment decisions such as glucocorticoids, antimicrobials, and/or vasoactive medications often precede diagnosis and may be counterproductive.

A 2018 study utilizing the French RespiRare Network found that nine of 34 pediatric patients with DAH of unknown etiology (i.e. idiopathic pulmonary hemosiderosis) from 1997 to 2017 also had T21, estimating a 75-fold higher prevalence in children with T21 [7]. Children with T21 had earlier onset, more severe respiratory distress, less hemoptysis, and increased morbidity and mortality. The reasons for this are neither well understood nor widely studied.

Here, we report five DAH cases in children with T21 at one center and a review of the literature. We aim to increase awareness of this life-threatening condition, highlight how it presents in this population, and identify opportunities to improve outcomes.

## Case presentations

Eleven children with DAH and T21 were identified in one institution’s histopathology database and summarized via chart review. Six were excluded due to disseminated intravascular coagulation, trauma, and/or perinatal causes. All five remaining patients were Caucasian. Additional details are listed below and in Table 1. Additionally, a literature review was conducted of all published cases.

**Table 1:** Case Descriptions

	Patient 1	Patient 2	Patient 3	Patient 4	Patient 5
**Presentation**					
**Sex**	Male	Female	Female	Female	Female
**Age at 1st known DAH (years)**	7	3	9	8	3
**Presenting Symptoms**	Fever, rash, joint pain, respiratory failure	Persistent oxygen requirement	Recurrent pneumonia and aspiration	Respiratory Distress, hypoxia	Hemoptysis
**Hemoptysis**	No	No	No	No	Yes
**Laboratory Result**^a^					
**White Blood Cell Count**	H	N	N	U	N
**Hemoglobin**	L	L	H	L	N
**Platelets**	L	N	N	U	N
**Inflammatory Markers**					
*Erythrocyte Sedimentation Rate*	H	H	N	U	N
*C-reactive Protein*	H	H	N	U	U
*Ferritin*	H	H	U	U	U
**Antibodies**^b^					
*Positive*	–	–	C-ANCA, MPO	MPO	ANA, SSA, RNP, Smith, Histone, CCP, RF
*Negative*	ANCA, ANA	–	ANA, Anti-GBM	ANCA, PR3, ANA	–
**Creatinine**	H	U	U	U	
**Investigations**^b^					
**Echocardiogram**	ND	Mild PAH	Mild PAH, AV regurgitation	Mild PAH, AV regurgitation and stenosis	ND
**Bronchoscopy with BAL**	ND	Bloody fluid return	Hemosiderin-Laden Macrophages	Bloody fluid return	Hemosiderin-Laden Macrophages
**Chest CT**	ND	Y	Y	Y	Y
*Ground Glass Opacities*	–	Y	Y	Y	Y
*Cystic Lucencies*	–	Y	Y	Y	Y
*Other Findings*	–	Atelectasis, small pleural effusions	Atelectasis, septal thickening	Diffuse centrilobular nodules, septal thickening	–
**Lung Biopsy**^c^	^d^				
*Alveolar Hemorrhage*	Mild	Mild	Marked	Mild	Moderate
*Hemosiderin*	Mild	Moderate	Marked	Marked	Minimal
*Abnormal Alveolar Growth*	Y	Y	Y	Y	Y
*Pulmonary Artery Thickening*	Moderate	Moderate	Moderate	Moderate	Moderate
*Other Findings*	Double capillary layer, focal interstitial fibrosis	Focal pneumonia, cholesterol clefts, subpleural type 2 cell proliferation	Airway Damage (repeat biopsy with plasma cell/CD3+ lymphocytes)	Double capillary layer, rare interstitial and pleural perivascular neutrophils	–
**Treatment**					
**Glucocorticoids**	Y	Y	Y	Y	Y
**Other Medications**	Antibiotics	Continued anakinra for SJIA	IVIG	IVIG, Rituximab	–
**Outcome**					
	Deceased	Remained on 0.5 L/min of oxygen via nasal cannula 3 months later, then lost to follow up	Repeat BAL with red blood cells 1 year later. Therapy stopped after 20 months. No recurrences since (6 years).	2 recurrences, both requiring ICU admission (one with Influenza B, one with Human Metapneumovirus). No recurrences since (1 year).	Recurrences through age 14. Glucocorticoids stopped with no known recurrences since (3 years).

*Abbreviations*: *DAH* Diffuse Alveolar Hemorrhage, *PAH* Pulmonary Arterial Hypertension, *AV* Atrioventricular Valve, *ANCA* Anti-neutrophil cytoplasmic antibody, *MPO* Anti-myeloperoxidase antibody, *PR3* Anti-serine protease 3 antibody, *BAL* Bronchoalveolar lavage, *SJIA* Systemic Juvenile Idiopathic Arthritis, *IVIG* Intravenous Immunoglobulin, *SSA* Anti-Sjogrens-Syndrome-related antigen A*, RNP* Anti-Ribonucleoprotein, *CCP* Anti-Cyclic-Citrullinated Peptide, *RF* Rheumatoid Factor, *ICU* Intensive Care Unit

^a^H = High, L = Low, N = Normal, U = Unknown

^b^Not listed or (−) = unknown

^b^Y = Yes, N = No, ND = Not Done

^c^All performed after exposure to glucocorticoids,

^d^Histopathology from autopsy

***Patient 1*** was born at 35 weeks gestation with a tracheoesophageal fistula and later developed asthma. He presented at age six with fever, rash, arthralgia, and severe anemia. Symptoms persisted until he died at age seven due to hemophagocytic lymphohistiocytosis, likely secondary to systemic juvenile idiopathic arthritis. Lung histology at autopsy revealed DAH, abnormal alveolar growth, moderate pulmonary artery thickening, a double capillary layer, and focal interstitial fibrosis.

***Patient 2*** was born at 40 weeks gestation with an atrioventricular septal defect (AVSD) and duodenal atresia. At age two, she developed systemic juvenile idiopathic arthritis managed with anakinra. She was admitted for hypoxia at age three. Chest computed tomography (CT) demonstrated subpleural cystic lucencies, ground glass opacities, atelectasis, and small pleural effusions. BAL showed acute inflammation and bloody fluid return. Lung biopsy revealed DAH, abnormal alveolar growth, moderate pulmonary artery thickening, focal pneumonia, cholesterol clefts, and subpleural type 2 cell proliferation. Echocardiogram demonstrated mild pulmonary arterial hypertension (PAH). She remained on anakinra and 0.5 L/min of oxygen 3 months later at which time she was lost to follow-up.

**Fig. 1:**
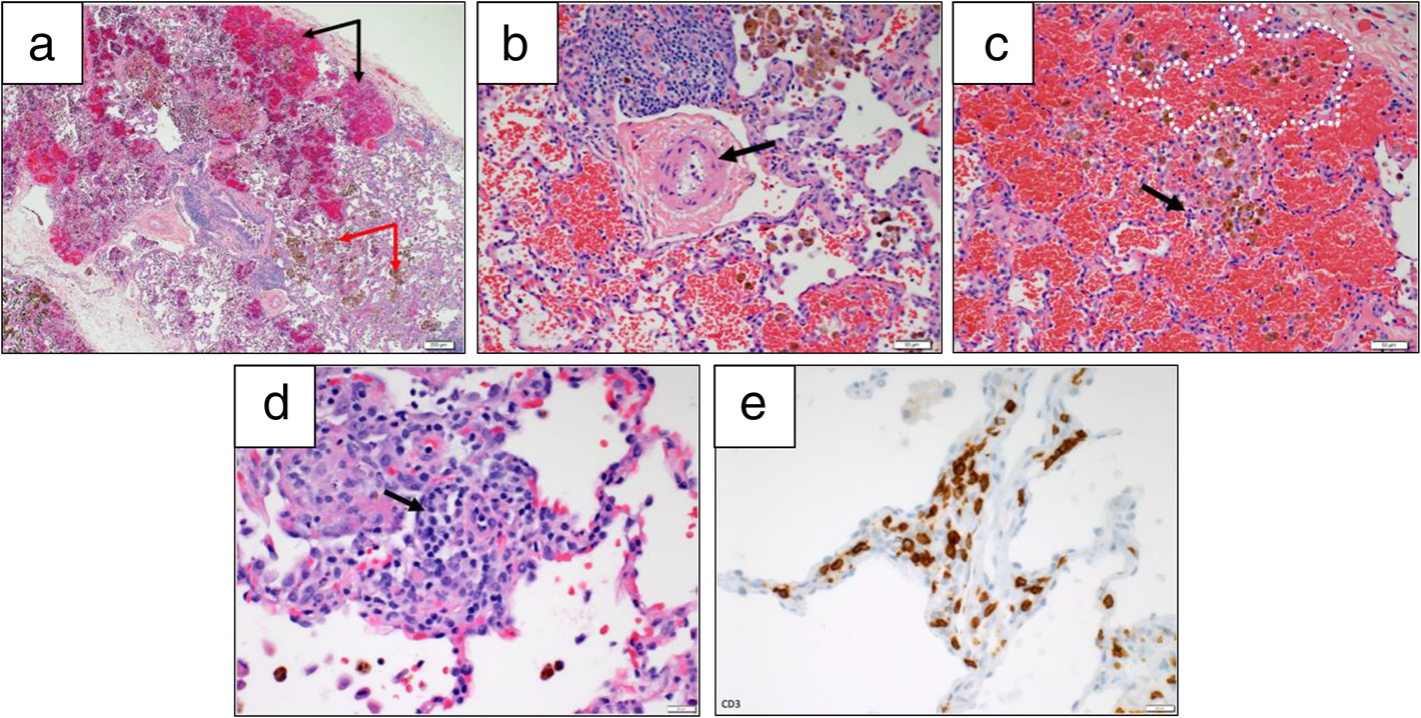
Lung biopsy findings in a 11-year-old female with trisomy 21 (Patient 3). **a** Low power view shows areas of alveolar hemorrhage (black arrows) and hemosiderin laden macrophages (white arrows). Hematoxylin-eosin stain, 4x. **b** High power view shows a moderately remodeled pulmonary artery (arrow points to pathologically muscularized arteriolar wall). Hematoxylin-eosin stain, 20x**. c** High power view shows hemorrhage, hemosiderin laden macrophages within simplified and distended alveoli (example is white dotted). A rare neutrophil (black arrow) is seen in the alveolar interstitium, not diagnostic of capillaritis. Hematoxylin-eosin stain, 20x. **d** The inflammatory infiltrate on repeat biopsy is mainly composed of lymphocytes, but rare plasma cells are noted (black arrow), 40x. **e** The majority of lymphocytes on repeat biopsy are marked by a T-cell immunomarker CD3, 40x

***Patient 3*** was born at 36 weeks gestation with a laryngeal cleft and AVSD. She presented for pulmonary evaluation at age nine due to recurrent suspected pneumonia, chronic aspiration, and obstructive sleep apnea (OSA). Chest CT demonstrated diffuse ground glass opacities, cystic lucencies, septal thickening, and atelectasis. BAL showed elevated hemosiderin-laden macrophages. Lung biopsy revealed DAH, abnormal alveolar growth, moderate pulmonary artery thickening, and airway damage (Fig. 1). She started monthly intravenous immunoglobulin (IVIG) infusions and daily glucocorticoids. Repeat lung biopsy after 6 months showed both plasma cells and CD3+ lymphocytes, raising suspicion for immune-mediated dysregulation. BAL after 12 months had persistent red blood cells. IVIG and glucocorticoids were stopped after 20 months without further DAH over the past 6 years.

**Fig. 2:**
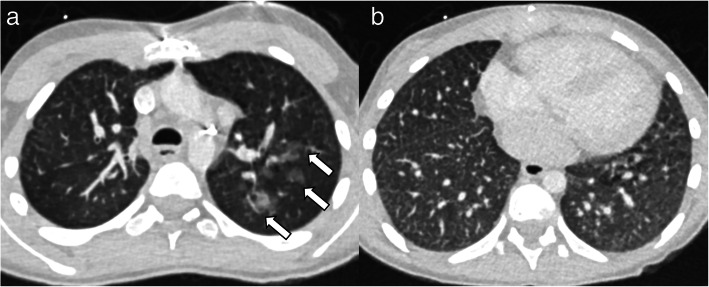
Chest CT findings in a 13-year-old female with trisomy 21 (Patient 4). Axial CT images through the upper (**a**) demonstrate ill-defined nodular ground glass opacities in the left upper lobe (arrows). Axial CT image through the lower chest (**b**) demonstrates innumerable tiny centrilobular nodules and septal thickening bilaterally

***Patient 4*** was born at 39 weeks gestation with an AVSD, galactokinase deficiency, prothrombin gene mutation, and left atrioventricular valve insufficiency. She developed OSA, sinusitis, and recurrent pneumonia in early childhood and underwent mitral valve repair at age eight. She presented with fatigue and pallor 1 month later and was diagnosed with DAH. At age nine, she presented to this hospital after a year of recurrent hemorrhages. Lung biopsy revealed DAH, abnormal alveolar growth, moderate pulmonary artery thickening, and a double capillary layer; rare interstitial and pleural perivascular neutrophils signaled possible resolving capillaritis. She received IV methylprednisolone and rituximab therapy followed by 2 years of IVIG and glucocorticoids. Repeat biopsy at 11 showed pulmonary hemosiderosis. At 13 and 16, she required ICU admission and IV glucocorticoids for DAH with influenza B and human metapneumovirus, respectively. She re-started rituximab during the second admission. Chest CTs demonstrated diffuse centrilobular nodules, ground glass opacities, septal thickening, and mild cystic lucencies (Fig. 2). BAL showed bloody fluid return. She remains on rituximab without recurrence of DAH 1 year later.

***Patient 5*** was born at 26 weeks gestation and developed bronchopulmonary dysplasia, aspiration, hypothyroidism, aortic and tricuspid valve insufficiency, and mild PAH. She developed recurrent hemoptysis at age three and began daily prednisone. Chest CT demonstrated diffuse ground glass opacities and cystic lucencies. BAL revealed hemosiderin-laden macrophages. Lung biopsy showed DAH, abnormal alveolar growth, and moderate pulmonary artery thickening. She remained on steroids for a decade due to recurrent hemoptysis. At 14, she presented to this hospital for evaluation. Steroids were discontinued. The next year, chest CT demonstrated diffuse ground glass opacities despite unremarkable BAL. Chest CT showed resolved opacities 1 year later with no known recurrences in the 3 years since.

***Review of the literature*** identified fewer than ten published, English-language cases of DAH in children with T21 outside of the RespiRare study and only one from the United States (Table 2) [8, 10–16, 21, 22]. As expected with Trisomy 21, the majority of children had cardiac anomalies, pulmonary hypertension, and/or structural lung abnormalities. Of the 19 total cases, the majority had anemia, respiratory distress, autoantibodies, and recurrences. A minority had hemoptysis. Idiopathic pulmonary hemosiderosis was the most common diagnosis. Almost all patients received glucocorticoids with or without other immunosuppression.

**Table 2:** Literature Review

Author, Year published	Sex	Ethnicity	Comorbidities in addition to T21	Age at 1st DAH (years)	Presenting Symptoms	Known Antibody Testing^a^	Diagnosis	Treatment	Outcome
**Alimi, 2018** [10]	4 F, 5 M	French	3 with PAH, 3 with cardiopathy	Mean: 2.92 +/− 3.45	2 with hemoptysis, all with dyspnea, 6 with hemoglobin < 7	6 with antibodies: ANCA, ANA, CCP, PR3, MPO, TTG-IgA, IgE-CMP	IPH	All: glucocorticoids 1: hydroxychloroquine 3: mycophenolate mofetil, azathioprine, and/or cyclophosphamide	3 without relapses 6 with relapses, of which 3 died
**Zhang, 2019** [11]	–	Chinese	–	–	1 of 107 children hospitalized with IPH at a single institution over a 21 year period	–	IPH	–	–
**Hori, 2015** [12]	F	Japanese	PAH, history of Kawasaki disease	2	Pallor, anorexia, dyspnea, hemoptysis, nephritis	+MPO	AAV	azathioprine, glucocorticoids	Improved, stable at time of publication
**Rayment, 2017** [13]	F	–	Repaired AVSD	2.5	Respiratory distress, anemia, thrombocytopenia	+ANCA	Isolated pulmonary capillaritis, bilateral pulmonary embolisms	glucocorticoids, rituximab, azathioprine	Improved, stable for at least 2.5 years
**Watanabe, 2015** [14]	F	Japanese	Hypothyroidism, autism	9	Cough, emesis of bloody sputum, hypoxemia, fatigue	+ANA, dsDNA	IPH	glucocorticoids	Recurred with hemoptysis 2 weeks later, then stable at least 4 months
**Galent, 1983** [15]	M	African American	PAH	2	Respiratory distress, blood-streaked sputum, fever, nasal discharge, cough	Elevated IgD antibody to milk proteins	Milk-related pulmonary hemosiderosis and cor pulmonale	Removal of cow’s milk from diet	Symptoms resolved. Re-introduction and removal repeated pattern.
**Aceti, 2012** [16]	F	Italian	History of VSD, ASD, PDA, necrotizing enterocolitis	4	Recurrent anemia and respiratory distress, hemoptysis	Negative ANA, ANCA, anti-GBM, TTG-IgA	Idiopathic Pulmonary Hemosiderosis	glucocorticoids	Recurred within 7 months, added hydroxychloroquine, recurred within 1 year
**Schwab, 1996** [17]	M	German	–	6	Recurrent pneumonia, hemoptysis, anemia, renal failure	+pANCA	AAV	glucocorticoids, cyclophosphamide	No recurrence in 2 years
**Niimi, 1992** [18]	F		VSD	17	Dyspnea	+ANA Negative RF, Anti-GBM		glucocorticoids	3 recurrences in 4 years
**Sato, 1986** [19]	F	Japanese	–	3	Cough, wheeze, anemia	–	–	azathioprine, disodium cromoglycate inhalation, milk avoidance	7 recurrences in 1 year
**Koyama, 1995** [20]	F	Japanese	–	9	Fever, dyspnea, anemia, pallor	–	IPH	glucocorticoids	No recurrence in 20 months

(−) = unknown

*Abbreviations*: *PAH* Pulmonary Arterial Hypertension, *ANCA* Anti-neutrophil cytoplasmic antibody, *ANA* Anti-nuclear antigen, *CCP* Anti-Cyclic Citrullinated Peptide Antibody, *PR3* Serine Protease 3 antibody, *MPO* Anti-Myeloperoxidase Antibody, *IgE-CMP* Immunoglobulin E to Cow’s Milk Protein, *IPH* Idiopathic Pulmonary Hemosiderosis, *AAV* ANCA-Associated Vasculitis, *AVSD* Atrioventricular Septal Defect, *dsDNA* Anti-double stranded DNA antibody, *IgD* Immunoglobulin D, *VSD* Ventricular Septal Defect, *ASD* Atrial Septal Defect, *PDA* Patent Ductus Arteriosus, *GBM* Glomerular Basement Membrane, *TTG-IgA* Tissue Transglutaminase Immunoglobulin A Antibody, *pANCA* Perinuclear ANCA, *RF* Rheumatoid Factor

^a^if unlisted, test is unknown

## Discussion and conclusions

These cases emphasize the variety of ways in which DAH presents in children with T21. The reasons for increased risk are multifactorial but likely include the following:

1. *Congenital cardio-pulmonary abnormalities* may increase risk of DAH in children with T21. Children with T21 have alveolar simplification, which is often compounded by prematurity. These structural lung and cardiac anomalies likely alter their propensity to develop DAH independent of other risk factors. Children with T21 are also at risk for vascular abnormalities, such as left-to-right shunts and persistent double capillary networks [17].
2. *PAH* may lead to DAH through increased hydrostatic pressure, vasodilator adverse effects, or aneurysmal rupture [18, 19]. At least 25% of children with T21 have PAH due to issues such as abnormal vascular walls, OSA, and heart disease [1]. Three of our patients had echocardiograms showing pulmonary hypertension and all had pulmonary artery thickening on histology.
3. *Infection and aspiration* may increase the risk for DAH and are more prevalent in children with T21 [20]. The similarity of radiographs and symptoms in these three conditions may lead to missed or delayed diagnosis of DAH.
4. *Autoimmune disease and immune dysregulation* may increase risk of DAH and are more prevalent in children with T21. Celiac disease, for instance, has a 6–10 fold higher frequency in children with T21 and is associated with increased risk of DAH [20]. Children with T21 have altered innate and adaptive immune responses and a higher prevalence of autoantibodies [7, 10, 11, 15, 23–26]. Within our cohort, one patient had secondary hemophagocytic histiocytosis, one had systemic JIA and three had autoantibodies of unknown significance.

Our data shows that DAH should be considered in children with T21 in the setting of respiratory distress, hypoxia, anemia, and/or ground glass opacities on imaging. Inflammatory markers may or may not be elevated and autoantibodies are often present. Hemoptysis was infrequent and cannot be relied on for diagnosis, while misdiagnosis of recurrent pneumonia often led to diagnostic delay. Diagnosis and management often involve both pulmonologists and rheumatologists.

Most patients within our cohort and the literature responded to immunomodulating therapies such as steroids, IVIG, and rituximab, supporting immune dysregulation as an etiology of DAH. Our patients lacked clear immune-mediated pulmonary capillaritis on lung histology; however, all samples were obtained after exposure to glucocorticoids and may have had an attenuated inflammatory response.

This series is limited by its retrospective focus at one institution; however, five patients over a 10-year period is a robust sample when compared to the literature [7]. Our hospital has a catchment area that includes an estimated 3.4 million children (personal communication, Children’s Hospital Colorado Marketing Office). Given an incidence of approximately 1 in 700 births, we would expect approximately 4800 of the children to have T21 [1]. Based on the French cohort estimates for DAH in T21, we would expect approximately six patients in our population with T21 to have DAH, consistent with our findings. However, given our hospital’s T21 specialty clinic only follows about one-third of the T21 patients in this multi-state area (~ 1600 of ~ 4800 predicted), the true prevalence of DAH may be even higher.

Underestimation of prevalence is also likely influenced by a low index of suspicion among providers. Many patients with T21 and respiratory symptoms do not undergo CT imaging, bronchoscopy, or lung biopsy and therefore DAH may be missed. Additionally, while these cases report the presence of autoantibodies and/or structural variations, it is not possible to confirm their direct role in DAH development. Further characterization of this population through a larger, multi-institutional sample will better define the spectrum of disease and guide care.

In summary, we recommend an increased index of suspicion for DAH among children with T21 who present with respiratory symptoms as they rarely present with hemoptysis. Providers should have a low threshold to evaluate for anemia and autoimmune pathology and consider pursuing additional imaging or procedures. Improved awareness of DAH in children with T21 will allow for earlier detection and reduce morbidity and mortality in this vulnerable population.

## Data Availability

Not Applicable.

## Abbreviations

T21: Trisomy 21
DAH: Diffuse Alveolar Hemorrhage
BAL: Bronchoalveolar lavage
AVSD: Atrioventricular septal defect
CT: Chest computed tomography
PAH: Pulmonary arterial hypertension
OSA: Obstructive sleep apnea
IVIG: Intravenous immunoglobulin
AV: Atrioventricular Valve
ANCA: Anti-neutrophil cytoplasmic antibody
MPO: Anti-myeloperoxidase antibody
PR3: Anti-serine protease 3 antibody
SJIA: Systemic Juvenile Idiopathic Arthritis
SSA: Anti-Sjogrens-Syndrome-related antigen A
RNP: Anti-Ribonucleoprotein
CCP: Anti-Cyclic-Citrullinated Peptide
RF: Rheumatoid Factor
ICU: Intensive Care Unit
ANA: Anti-nuclear antigen
IgE-CMP: Immunoglobulin E to Cow’s Milk Protein
IPH: Idiopathic Pulmonary Hemosiderosis
AAV: ANCA-Associated Vasculitis
dsDNA: Anti-double stranded DNA antibody
IgD: Immunoglobulin D
VSD: Ventricular Septal Defect
ASD: Atrial Septal Defect
PDA: Patent Ductus Arteriosus
GBM: Glomerular Basement Membrane
TTG-IgA: Tissue Transglutaminase Immunoglobulin A Antibody
pANCA: Perinuclear ANCA
